# Pain-side-specific alteration of structural networks in trigeminal neuralgia: a connectome analysis

**DOI:** 10.3389/fnins.2026.1794457

**Published:** 2026-05-29

**Authors:** Xiao-Yi Guo, Yongtao Zheng, Lin-Qing Zhao, Hanbing Shang, Wenlei Yang

**Affiliations:** 1 Department of Radiology, Ruijin Hospital, Shanghai Jiaotong University School of Medicine, Shanghai, China; 2 Department of Neurosurgery, Ruijin Hospital, Shanghai Jiaotong University School of Medicine, Shanghai, China

**Keywords:** brain structural network, diffusion tensor imaging, graph theoretical analysis, network-based statistics, trigeminal neuralgia

## Abstract

**Objectives:**

Trigeminal neuralgia (TN) involves disruption in the integrity of the white matter, the side-specific pain topology of these alterations at the network has yet to be defined. In this study, we investigated the lateralization of structural network architecture and nodal characteristics in TN patients.

**Methods:**

Whole-brain structural networks (90 × 90 connectivity matrices) were reconstructed from diffusion tensor imaging (DTI) tractography data of 30 TN patients and 20 matched controls. We applied Network-Based Statistics (NBS) to detect altered connectivity sub-networks, and graph theoretical analysis to profile global and nodal properties. Our analysis aimed to delineate changes that were specific to the painful side.

**Results:**

NBS analysis revealed that structural connectivity formed subnetworks involving multiple functional networks. A subnetwork involving the anterior cingulate gyrus (ACG) and postcentral gyrus (S1) was identified on the painful side, indicating that TN stimulation may enhance structural connectivity between regions related to salience and somatosensory processing, thereby facilitating the acceleration of pain perception and response. On the non-pain side, we observed enhanced structural connections between visual and attention-related regions. The third subnetwork was characterized by widespread and non-focal reductions in fiber tract connectivity. However, despite these localized alterations, the global network properties of the brain in TN patients remained stable, with node-specific properties undergoing alterations in multiple brain regions, including the cuneus, inferior parietal lobule, and superior frontal gyrus.

**Conclusion:**

Herein, we applied NBS and graph theoretical analysis to investigate changes in the structural brain networks of patients with TN. Analysis revealed that specific subnetworks and key nodes can be affected by TN. We also confirmed obvious differences in the involved subnetworks between pain and non-pain sides in TN patients. These findings suggest that these specific subnetworks and nodes could represent valuable biomarkers for clinical evaluation and intervention in TN patients.

## 1 Introduction

Classic trigeminal neuralgia (TN) is traditionally conceptualized as arising from vascular compression of the trigeminal nerve at the root entry zone (REZ) and the consequent morphological changes (Headache Classification Committee of the International Headache Society (IHS), 2018). Histological studies have consistently demonstrated the presence of white matter demyelination and axon swelling at the nerve root in TN (Beaver, 1967). However, recent neuroimaging evidence suggests that TN-related abnormalities are not confined to the REZ, but may involve more widespread structural and functional alterations within the brain (Nardoni et al., 2025; Xu et al., 2022). To map brain injury and its subsequent reconstruction in a non-invasive manner, and to characterize alterations in both the intracortical and interhemispheric pathways under disease burden, researchers applied connectomics to conceptualize the whole brain as an interconnected network, thus enabling the delineation of structural connectivity patterns across the human brain (Hagmann et al., 2008).

Network-based statistics (NBS) and graph theory are widely used methodologies in connectomics and are particularly useful for characterizing complex network alterations associated with neurological disorders and chronic pain conditions (Butry et al., 2025). In particular, NBS is designed to identify clusters of interconnected edges (i.e., connected components or subnetworks) that show statistically significant group differences, enabling the detection of disease-related connectivity alterations (Zalesky et al., 2010). Graph-theoretical measures can be broadly categorized based on their functional significance, including measures of integration, segregation, centrality, and resilience as proposed by Rubinov and Sporns (2010). Recent work has further emphasized the importance and measurement properties of integration and segregation-related network indices in brain network research (Han et al., 2024). Measures of integration, such as global efficiency, quantify the capacity for parallel information transfer across the brain network, reflecting the efficiency of distributed communication. In contrast, measures of segregation, including clustering coefficient and small-world properties, characterize the degree of specialized processing within densely interconnected local regions. Node-level measures, such as degree and betweenness centrality, capture the relative importance or influence of specific brain regions within the network. Within this framework, graph theory provides a comprehensive approach for examining both global and local topological properties of brain networks, thereby facilitating a more systematic evaluation of network organization at multiple levels.

The methodological advances described above have been applied in numerous studies examining abnormalities in whole-brain structural and functional networks in TN. Within this framework, prior studies have reported alterations across multiple dimensions of network organization, including integration, segregation, and centrality. Using resting-state functional magnetic resonance imaging (rs-fMRI) and dynamic functional network connectivity (dFNC), Zhang et al. (2021) reported disrupted global efficiency and small-world properties in TN, suggesting impaired network integration. More recent multimodal neuroimaging studies have further demonstrated abnormalities in both structural and functional topological organization in TN. Wang et al. (2025) reported altered small-world properties, reduced nodal efficiency, and disrupted limbic functional connectivity in patients with classical TN. Huang et al. (2025) further integrated rs-fMRI and diffusion tensor imaging (DTI) to examine whole-brain functional connectivity, topological properties, and structural-functional coupling, revealing disrupted structural and functional topological organization within specific subnetworks in classical TN. In addition, connectome-based analyses further support the view that TN involves distributed brain network dysfunction rather than an isolated peripheral lesion (Chu et al., 2025). Collectively, these findings suggest that TN is associated with widespread disruptions in brain network organization, yet the hemispheric lateralization of structural connectivity abnormalities has not been fully characterized.

In the present study, we aimed to analyze the brain networks in the affected and unaffected hemispheres of TN patients by DTI. By applying NBS, we investigated whether structural connectivity components exhibited lateralized patterns. First, we segregated the nodes into the affected and unaffected hemispheres to investigate network connectivity and laterality-associated alterations in the brain networks of TN patients. Then, we employed graph-theoretical metrics to characterize global network organization and nodal characteristics.

## 2 Methods

### 2.1 Participants

Between January 2024 and July 2025, a total of 34 patients with TN and 23 healthy controls (HCs) were consecutively recruited from the Department of Neurosurgery. This cohort included individuals diagnosed with classical TN.

The inclusion criteria for the patient group were as follows: (1) a diagnosis of TN based on the International Classification of Headache Disorders, 3rd edition (ICHD-3); (2) evidence of neurovascular compression, identified on preoperative magnetic resonance imaging texture analysis (MRTA) by visualization of vascular contact with, displacement of, or distortion of the trigeminal nerve root, and subsequently confirmed intraoperatively; and (3) MRI signs of small vessel disease with a Fazekas score ≤1. This criterion was applied to reduce the potential confounding effects of white matter hyperintensities on diffusion metrics and structural network topology.

The exclusion criteria were as follows: (1) unstable general health status (e.g., a history of cerebrovascular events, previous neurosurgical procedures, or the presence of neurological or psychiatric disorders); (2) previous treatment with Gamma knife radiosurgery (GKRS) or any other form of cranial surgery; and (3) the presence of metal implants. HCs were recruited from the general population; the inclusion criteria for HCs were as follows: no history of headache, facial pain, or neurological disorders. All participants underwent high-resolution MRI to exclude space-occupying lesions, such as tumors. Four TN patients were excluded from the final analysis due to severe motion artifacts (*n* = 2) and high Fazekas scores (*n* = 2; Fazekas score = 2), and three HCs were excluded due to high Fazekas scores (Fazekas score = 2). Consequently, a total of 30 TN patients and 20 HCs were included in the final analysis.

The study was approved by the Institutional Review Board (IRB) of Ruijin Hospital, Shanghai Jiao Tong University School of Medicine. Written informed consent was obtained from all participants or their legal guardians after a full explanation of the procedures.

### 2.2 Pain intensity assessment

Pain severity was assessed using the Barrow Neurological Institute (BNI) pain intensity score, a categorical scale ranging from grade I to V (Rogers et al., 2000). Detailed grading criteria are provided in Supplementary Table 1.

### 2.3 MRI data acquisition and pre-processing

Diffusion imaging was acquired using a 3.0 T clinical scanner (Ingenia, Philips Healthcare, Best, Netherlands). DTI sets were acquired with b = 0 and b = 800 s/mm^2^ (Echo planar, TR = 8,084 ms, TE = 84 ms, flip angle = 90°, MPG = 64 directions, FOV = 192 mm, matrix = 96 × 96, slice thickness = 2 mm, with posterior–anterior phase encoding orientation). One additional image was acquired without diffusion weighting (i.e., b = 0 s/mm^2^) with the same acquisition parameters as above, except phase encoding orientation was converted to the anterior–posterior. Conventional morphology T1-weighted images were also acquired with the following parameters: TR = 6,746 ms, TE = 3.0 ms, flip angle = 8°, FOV = 240 × 240 mm, matrix = 1 × 1, slice thickness = 1 mm.

### 2.4 MRI data pre-processing

Image preprocessing was performed using the diffusion toolbox of the functional magnetic resonance imaging of the brain (FMRIB) software library (FSL) (Woolrich et al., 2009). Image preprocessing included three steps. First, “topup” was used to estimate the susceptibility-induced off-resonance field (Smith et al., 2004). Next, we performed “eddy current and motion correction’ to correct image distortion and head motion artifacts (Andersson and Sotiropoulos, 2016). Finally, “dtifit” was used to fit the diffusion tensor model at each voxel, generating the tensor images.

### 2.5 Whole brain connectivity

The diffusion tensor model was processed using the Pipeline for Analyzing Brain Diffusion Images (PANDA) toolkit^1^. Deterministic fiber tracking was performed using the Fiber Assignment by Continuous Tracking (FACT) algorithm based on the fractional anisotropy (FA) maps, and fiber number (FN) matrices were constructed by counting the number of reconstructed streamlines between each pair of regions. The resulting connectivity matrices were undirected and symmetric, with FN(i,j) = FN(j,i). Tracking was conducted with the following parameters: angle threshold = 45°, step length = 1 mm, FA threshold = 0.2, and a spline filter to smooth the fiber trajectories. The MNI152 T1 brain template was used for anatomical registration to align individual subject data into standard space. The brain was then parcellated using the Automated Anatomical Labeling (AAL90) atlas, a widely used and well-validated parcellation scheme in structural connectome studies that provides a balance between anatomical interpretability and network resolution (Bullmore and Sporns, 2009; Tzourio-Mazoyer et al., 2002) (node names and corresponding abbreviations are detailed in Supplementary Table 2). FN matrices, representing the structural connectivity between regions, were constructed from the results of deterministic fiber tracking. The resulting connectivity matrices were undirected and symmetric, with FN(i,j) = FN(j,i). These 90 × 90 FN matrices were used as the basis for both NBS and graph-theoretical analyses. NBS was applied at the edge level to identify subnetworks showing group differences. Graph-theoretical metrics were computed from the full connectivity matrices for each subject, including both global and nodal measures. Then, the nodes were divided into pain and non-pain sides, and the FN matrices were recombined accordingly. Specifically, to integrate patients with left and right sided pain, we employed a side-flipping strategy. The left hemisphere was defined as the “pain side” and the right hemisphere as the “non-pain side.” For left-sided pain patients, their original left–right orientation was preserved. For right-sided pain patients, structural connectivity matrices were mirrored, re-mapping pain-related regions from the right hemisphere to the left (pain side) and non-pain regions from the left hemisphere to the right (non-pain side). The control group underwent no mirroring, with their left and right hemispheres compared to the pain and non-pain sides of the patients. NBS and graph-theoretical network analysis were subsequently repeated.

### 2.6 Network-based statistics (NBS) analysis

NBS was employed to identify significant group differences in structural brain networks between TN patients and HCs. Connectivity between predefined brain regions was assessed using edge-by-edge two-sample t-tests. A primary threshold of *t* = 2.1 was applied to the edge-wise between-group t statistics to define suprathreshold edges. Connected components (i.e., subnetworks) formed by these suprathreshold edges were identified, and statistical inference was performed at the level of these components using permutation testing to control for multiple comparisons. To further characterize the NBS thresholding procedure, suprathreshold node-to-node edges identified from the edge-wise *t*-statistic matrix at the primary threshold of *t* = 2.1 in both directional contrasts were matched to the original PANDA FN matrices, and their raw FN values were summarized in Supplementary material. The dataset was randomly shuffled 5,000 times, with subjects reassigned to either of the two groups for each permutation. For each permutation, the largest subnetwork was identified, and its size was compared to that of the observed subnetwork in the original data. Statistical significance was determined using a family-wise error (FWE) corrected threshold of *p* < 0.05 across all subnetworks, as implemented in the standard NBS framework (Zalesky et al., 2010).

### 2.7 Global and regional network measures

We constructed structural brain networks and analyzed them as undirected, symmetric, binary (unweighted) graphs. Specifically, connectivity matrices were thresholded across a predefined proportional sparsity range (0.10–0.40, step = 0.01), with 10–40% of the strongest connections retained at each threshold, and the resulting matrices were binarized at each sparsity level. To reduce bias associated with single-threshold selection, graph metrics were integrated across the threshold range using the area under the curve (AUC) approach (Tsai, 2018). To further characterize the thresholding procedure, we summarized the pre-binarization FN values of retained edges across the nominal sparsity thresholds, as detailed in Supplementary material. For regional network properties, we assessed betweenness centrality, degree centrality, nodal clustering coefficient, nodal efficiency, nodal local efficiency, and nodal shortest path. For global network properties, we evaluated small-worldness and network efficiency, including both local efficiency and global efficiency as global network measures. For each subject and each network metric, AUC across the predefined sparsity range was calculated using the built-in GRETNA procedure as a threshold-independent summary measure (Wang et al., 2011; Wang J. et al., 2015). These AUC values were then used for between-group comparisons using non-parametric permutation testing with 1,000 permutations. Group labels were randomly reassigned while preserving sample sizes to generate an empirical null distribution under the null hypothesis of no group effect. Multiple comparisons across nodes were corrected using the Benjamini-Hochberg false discovery rate (FDR, *q* < 0.05). In addition, we also report Cohen’s d effect sizes for these node properties. Subsequently, the significant findings from the subnetwork connectivity and graph theory analyses were visualized with BrainNet Viewer (Xia et al., 2013).

To explore whether the primary nodal findings were disproportionately driven by patients with the highest pain severity, we performed an exploratory subgroup analysis in which TN patients were stratified by BNI pain intensity score (BNI grades 3–4 vs. BNI grade 5). As an exploratory analysis, TN patients were stratified by BNI pain intensity score (grades 3–4 vs. 5). Nodal measures showing significance in the primary nodal analysis were subsequently evaluated across HC, TN with BNI grades 3–4, and TN with BNI grade 5 using Kruskal–Wallis tests followed by pairwise Wilcoxon rank-sum tests. Uncorrected *p* values were reported because of the exploratory nature of this analysis.

### 2.8 Statistical analysis

Summary statistics for clinical demographic features are expressed as mean ± standard deviation. Group comparisons were conducted using Student’s *t*-tests. Duration is presented as median with range and interquartile range, respectively. Gender differences were assessed using Fisher’s exact test. The significance level for all statistical tests was set at *p* < 0.05.

## 3 Results

### 3.1 Demographic and clinical characteristics

The study cohort comprised 30 patients with TN and 20 HCs, matched for both age and gender. The mean age of participants was 60.5 ± 10.3 years in the TN group and 60.4 ± 7.7 years in the HCs group (*p* = 0.677). Gender distribution did not differ significantly when compared between the two groups (*p* = 0.071). Among patients with TN, the median disease duration was 42 months (IQR: 24–74.25 months). The median BNI pain intensity score was 4 with a range of 3–5 (Table 1).

**Table 1:** Demographic and clinical characteristics of the participants.

Variables	HCs (*n* = 20)	TN (*n* = 30)	*p*-value
Age	60.4 ± 7.7	60.5 ± 10.3	0.677
Gender (female/male)	*F* = 10, M = 10	*F* = 23, M = 7	0.071
Disease duration (mths)	–	42 (IQR: 24–74.25)	–
Pain laterality	–	L: 13, R: 17	–
Pain distribution	–	V1: 2, V1–2: 3, V2: 7, V2–3: 9, V3: 9	–
Pain intensity (BNI)	–	grades 3: 9, grades 4: 14, grades 5: 6	–

BNI, barrow neurological institute pain intensity score; F, female; M, male. Data are presented as mean ± SD or median (median and interquartile range) where appropriate.

### 3.2 Whole-brain mapping of connections

**Figure 1:**
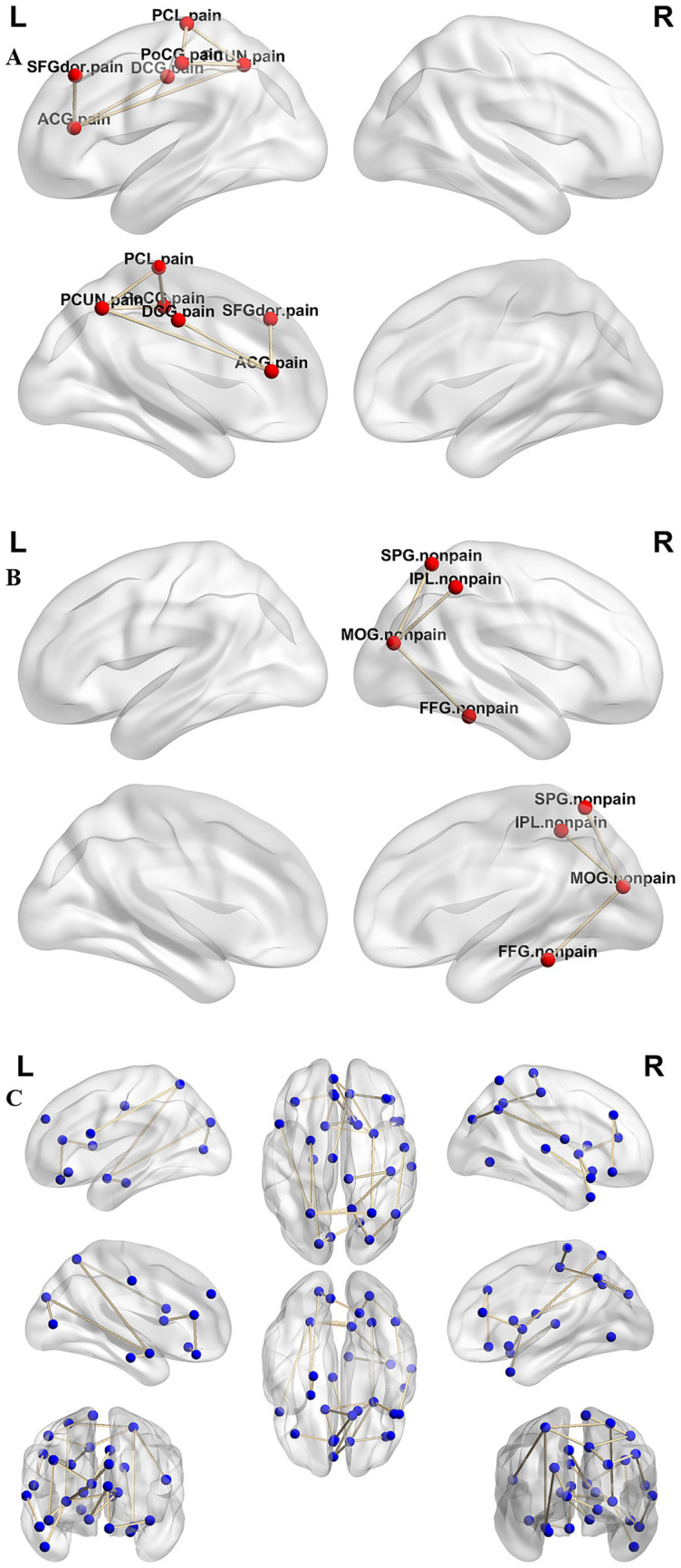
Summary of significant networks that characterize trigeminal neuralgia using NBS. The three subnetworks were observed. **(A)** Network 1 (6 nodes, 6 edges) mainly involves regions anatomically corresponding to the frontoparietal control, salience, somatomotor, and default-mode systems on the painful side. **(B)** Network 2 (4 nodes, 3 edges) mainly involves regions anatomically corresponding to the visual network and dorsal attention network on the non-painful side. **(C)** Network 3 (30 nodes, 29 edges) mainly involves regions anatomically corresponding whole brain.

NBS analysis revealed three subnetworks that differed significantly between TN patients and HCs (*p* < 0.05, FWE-corrected). In the first identified subnetwork, TN patients exhibited increased connectivity in the standardized painful hemisphere (aligned to the left hemisphere after side-flipping) compared to the corresponding hemisphere in healthy controls. This network comprises six edges linking six distinct regions (*p* = 0.006; Figure 1A). This identified subnetwork encompassed six nodes located in the affected hemisphere, including the dorsal superior frontal gyrus (SFGdor), anterior cingulate gyrus (ACG), median cingulate and paracingulate gyri (DCG), postcentral gyrus (PoCG), precuneus (PCUN), and paracentral lobule (PCL). These regions were interconnected by six edges spanning the frontal, cingulate, and parietal cortices.

Next, we identified a second subnetwork. TN patients exhibited an increased number of reconstructed tracts on the non-painful side compared to the painful side. This network comprised three edges linking four distinct regions (*p* = 0.029; Figure 1B). This identified subnetwork encompassed four nodes located in the non-painful hemisphere, including the middle occipital gyrus (MOG), fusiform gyrus (FFG), superior parietal gyrus (SPG), and inferior parietal lobule (IPL). These regions were interconnected by three edges, forming a distributed network spanning the occipital and parietal cortices.

In the third subnetwork, TN patients showed fewer reconstructed tracts on both the painful and non-painful sides when compared with HCs. This network comprises 29 edges linking 30 distinct regions (*p* = 0.014; Figure 1C). The affected nodes were distributed across the frontal, temporal, parietal, and occipital lobes, the cingulo-limbic system, and the striatum. A complete list of nodes is provided in Table 2.

**Table 2:** NBS subnetwork between TN patients and HCs and corresponding raw fiber-number values of component edges.

Networks and connections					
Edge^*^	Node 1	Node 2	HCs	TN	*t* and *p*-value
Network 1					
1	SFGdor.pain	ACG.pain	0.000 ± 0.000	2.300 ± 3.631	*t* = 2.82, *p* = 0.007
2	ACG.pain	DCG.pain	32.450 ± 18.392	45.300 ± 19.839	*t* = 2.31, *p* = 0.025
3	ACG.pain	PCUN.pain	3.600 ± 4.512	10.533 ± 13.310	*t* = 2.24, *p* = 0.030
4	PoCG.pain	PCUN.pain	0.250 ± 0.550	4.400 ± 7.005	*t* = 2.63, *p* = 0.011
5	PoCG.pain	PCL.pain	1.150 ± 1.531	7.667 ± 8.519	t = 3.37, *p* = 0.001
6	PCUN.pain	PCL.pain	5.350 ± 5.224	12.300 ± 10.920	*t* = 2.65, *p* = 0.011
Network 2					
1	MOG.nonpain	FFG.nonpain	7.700 ± 9.217	18.700 ± 20.395	*t* = 2.55, *p* = 0.014
2	MOG.nonpain	SPG.nonpain	16.750 ± 13.062	31.900 ± 22.913	*t* = 2.94, *p* = 0.005
3	MOG.nonpain	IPL.nonpain	0.650 ± 2.033	4.267 ± 5.813	*t* = 3.15, *p* = 0.003
Network 3					
1	MFG.nonpain	ORBinf.nonpain	2.000 ± 4.600	0.033 ± 0.183	*t* = 2.35, *p* = 0.023
2	ORBinf.pain	REC.pain	12.250 ± 12.135	6.033 ± 6.122	*t* = 2.39, *p* = 0.021
3	OLF.nonpain	ACG.pain	1.900 ± 3.478	0.400 ± 0.814	*t* = 2.28, *p* = 0.027
4	REC.pain	ACG.pain	4.550 ± 5.708	0.733 ± 1.388	*t* = 3.53, p = 0.001
5	MFG.nonpain	ACG.nonpain	0.850 ± 2.134	0.000 ± 0.000	*t* = 2.19, *p* = 0.033
6	SFGmed.pain	ACG.nonpain	11.750 ± 19.147	2.200 ± 5.635	*t* = 2.58, *p* = 0.013
7	ACG.nonpain	DCG.pain	1.300 ± 3.147	0.033 ± 0.183	*t* = 2.21, *p* = 0.032
8	PHG.pain	AMYG.pain	16.400 ± 9.544	9.267 ± 9.555	*t* = 2.59, *p* = 0.013
9	CAL.pain	LING.nonpain	1.900 ± 3.354	0.267 ± 0.740	*t* = 2.59, p = 0.013
10	AMYG.pain	SOG.pain	0.150 ± 0.366	0.000 ± 0.000	*t* = 2.25, *p* = 0.029
11	CAL.pain	SOG.pain	19.250 ± 11.867	11.767 ± 9.104	*t* = 2.52, *p* = 0.015
12	IFGoperc.pain	SPG.pain	0.250 ± 0.639	0.000 ± 0.000	*t* = 2.16, *p* = 0.036
13	AMYG.pain	SPG.pain	0.250 ± 0.639	0.000 ± 0.000	*t* = 2.16, p = 0.036
14	SPG.pain	SPG.nonpain	1.050 ± 2.212	0.100 ± 0.403	*t* = 2.31, p = 0.025
15	ROL.nonpain	ANG.nonpain	2.350 ± 4.804	0.400 ± 1.102	*t* = 2.15, *p* = 0.037
16	SOG.nonpain	ANG.nonpain	9.500 ± 9.736	3.800 ± 6.703	*t* = 2.46, *p* = 0.018
17	SOG.nonpain	PCUN.nonpain	14.550 ± 8.703	8.233 ± 5.594	*t* = 3.13, *p* = 0.003
18	PoCG.nonpain	PCUN.nonpain	4.500 ± 4.383	1.633 ± 4.056	*t* = 2.37, *p* = 0.022
19	SPG.pain	PCUN.nonpain	14.750 ± 22.332	3.033 ± 10.572	*t* = 2.49, *p* = 0.016
20	PoCG.nonpain	PCL.nonpain	11.850 ± 8.756	4.933 ± 7.423	*t* = 3.00, *p* = 0.004
21	OLF.nonpain	CAU.pain	2.150 ± 2.661	0.800 ± 1.215	*t* = 2.43, *p* = 0.019
22	ACG.pain	CAU.pain	10.000 ± 7.049	5.633 ± 5.857	*t* = 2.38, *p* = 0.021
23	SFGmed.pain	CAU.nonpain	1.700 ± 3.045	0.067 ± 0.254	*t* = 2.94, *p* = 0.005
24	ACG.nonpain	PUT.nonpain	0.700 ± 1.750	0.000 ± 0.000	*t* = 2.20, *p* = 0.032
25	SPG.nonpain	PUT.nonpain	17.350 ± 22.528	6.400 ± 8.728	*t* = 2.41, *p* = 0.020
26	CAU.pain	PUT.nonpain	0.400 ± 0.995	0.000 ± 0.000	*t* = 2.21, *p* = 0.032
27	CAU.nonpain	PUT.nonpain	21.600 ± 16.210	11.700 ± 7.966	*t* = 2.87, *p* = 0.006
28	PUT.nonpain	TPOsup.nonpain	10.000 ± 15.980	1.567 ± 2.800	*t* = 2.84, *p* = 0.007
29	STG.nonpain	TPOsup.nonpain	35.000 ± 18.169	24.667 ± 15.323	*t* = 2.17, *p* = 0.035
30	PUT.nonpain	TPOmid.nonpain	2.000 ± 2.428	0.367 ± 0.999	*t* = 3.30, *p* = 0.002

^*^The edge refers to the connection between node 1 and node 2.

### 3.3 Analysis of network characteristics

No significant between-group differences were observed across global network properties. Specifically, global and local efficiency did not differ significantly between groups, nor did small-world characteristics, including the clustering coefficient, characteristic path length, normalized clustering coefficient (*γ*), normalized path length (*λ*), and the small-worldness index (*σ*) (all *p* > 0.05); effect sizes were consistently small (|Cohen’s d| < 0.5). A complete dataset is provided in Supplementary Table 3.

**Figure 2:**
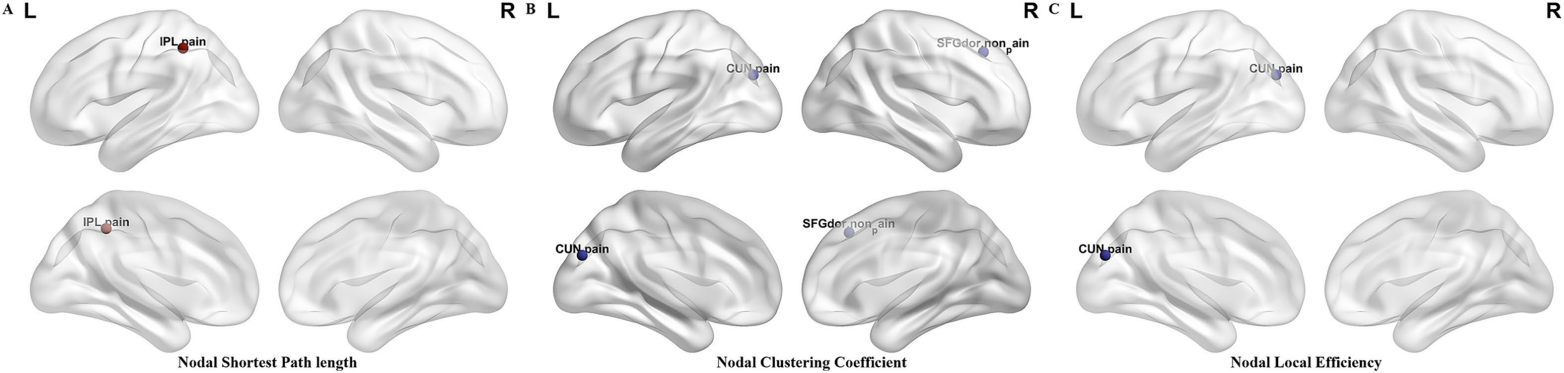
Brain regions showing between-group differences in nodal graph-theoretical measures, visualized using the AAL-90 atlas. Blue nodes represent regions where HCs > TN, whereas red nodes represent regions where HCs < TN. The AAL-90 atlas was used for visualization. **(A)** Nodal shortest path length; **(B)** Nodal clustering coefficient; and **(C)** Nodal local efficiency.

Figure 2 summarizes nodal properties across multiple network measures. With respect to nodal shortest path length, TN patients exhibited a significant increase at IPL.pain compared with HCs (pFDR = 0.035, |Cohen’s *d*| = 1.12). TN patients exhibited a significant decrease in the nodal clustering coefficient at SFGdor.non_pain (pFDR = 0.036, |Cohen’s *d*| = 0.98) and the cuneus on the pain side (CUN.pain; pFDR = 0.012, |Cohen’s *d*| = 1.11), compared with HCs. In addition, TN patients exhibited a significant decrease in nodal local efficiency at CUN.pain compared with HCs (pFDR = 0.027, |Cohen’s *d*| = 1.11). No significant differences were observed in degree centrality, betweenness centrality, or nodal efficiency after FDR correction. However, as part of an exploratory analysis, results that did not survive FDR correction, together with their corresponding effect sizes, are presented in Supplementary Table 4 and Supplementary Figure 1.

In the exploratory severity-stratified analysis, no significant differences were observed between TN patients with BNI grades 3–4 and those with BNI grade 5 across any of the nodal measures that showed significant differences in the primary analysis (Supplementary Figure 2).

## 4 Discussion

In this study, we utilized NBS and graph-theoretical methods to characterize alterations in brain microstructure in TN form both lateralization and topological perspectives. First, we identified three subnetworks showing significant differences between TN patients and HCs. Among them, two subnetworks demonstrated opposite lateralized connectivity patterns. One associated with the pain side, involving the frontal-cingulate-parietal circuit, and the other associated with the non-pain side, encompassing the temporal–parietal-occipital circuit. The third subnetwork showed widespread bilateral connectivity reductions. Second, while global network properties, including efficiency, small-world index, path length, and clustering coefficient, remained largely stable, significant alterations were observed at the nodal level. These findings align with recent neuroimaging evidence in chronic pain, suggesting that the overall small-world architecture remained preserved (Lenoir et al., 2021), while distributed, lateralized hub reweighting occurred.

The first pain-side subnetwork involved the dorsolateral superior frontal gyrus, ACG, median cingulate and paracingulate gyri, postcentral gyrus, precuneus, and paracentral lobule. This pattern indicates altered structural connectivity in TN, with enhanced coupling among regions associated with the salience network (SN), default mode network (DMN), executive control network, and sensorimotor network. Among these regions, the ACG is particularly noteworthy because it is a core component of the SN and plays an important role in pain-related attention, emotional processing, and descending pain modulation (Yang and Chang, 2019; Mercer Lindsay et al., 2021). In previous intracranial human studies, the ACG has been implicated in pain processing at multiple levels. Early single-neuron recordings identified pain-related neuronal responses in the human cingulate cortex (Hutchison et al., 1999), while more recent ambulatory intracranial recordings showed that acute evoked pain was associated with relatively transient ACG -related activity, in contrast to the more sustained orbitofrontal cortex signals that better tracked chronic spontaneous pain (Shirvalkar et al., 2023). Together, these findings support the view that the anterior cingulate region is centrally involved in rapid pain-state processing and salience-related behavioral adjustment. In our study, the ACG region also appeared to function as a pivotal node within the pain-side subnetwork, with a higher degree than other nodes and with connections bridging multiple network components. By linking regions related to the default mode, salience, and executive control systems, the ACG may facilitate efficient integration of attentional and executive resources in response to nociceptive input, thereby promoting rapid pain-related regulation. Moreover, the increased structural connectivity involving the postcentral gyrus, paracentral lobule, and precuneus suggests pain-lateralized interaction between sensorimotor processing and self-referential systems. The postcentral gyrus is the primary hub for sensory input to the primary somatosensory cortex (S1) and plays a critical role in processing the sensory-discriminative aspects of pain, serving as a core site at which thalamic pain information is integrated after reaching the cortex (Peyron et al., 2000). The adjacent paracentral lobule has also been implicated in enhanced responses to self-relevant pain-related stimuli (Khatibi et al., 2023). As a core hub of the DMN, the precuneus forms structural connections with the prefrontal cortex, supplementary motor area, and insular cortex through multiple anterior–posterior white matter pathways, thus supporting its role in mediating perceptual integration, attentional regulation, and actinon preparation (Dadario and Sughrue, 2023; Yamaguchi and Jitsuishi, 2024). Additionally, the activity pattern of the precuneus within the para-cingulate network indicates that this region is strategically positioned to integrate both internal and external stimuli, linking these stimuli with prior experiences to guide subsequent behavior, thereby facilitating processes such as the interpretation of sensory information and action selection in complex contexts (Lyu et al., 2021). Together, these findings suggest that the enhanced coupling observed in TN may reflect a shift toward a pain perception-action preparation pathway in chronic pain states, potentially shortening the transition from sensory representation of pain to behavioral readiness.

On the non-pain side, increased structural connectivity was observed among regions associated with the visual network and dorsal attention network (DAN), with the middle occipital gyrus appearing as a central node. This pattern suggests altered structural organization in the central nervous system on the non-pain side, particularly in terms of coordination of the visual-attentional pathways. Our findings may provide structural support for previously reported increases in functional connectivity between the visual network (VN) and DAN (Zhou et al., 2024), suggesting that these functional alterations could be accompanied by changes in white matter connectivity. This structural-functional coupling emphasizes the critical role of white matter integrity in supporting the functional network alterations associated with chronic pain. In addition to its traditional role in visual information processing, the VN is also involved in the assessment of pain salience and the modulation of visual attention. Previous work has shown that pain can disrupt normal visual exploration, including reduced eye-movement frequency and increased saccadic latency (Schmidt et al., 2018). Pain has also been associated with attentional bias, reflected by more frequent gaze shifts toward the ipsilateral visual field. Given the contralateral organization of visuospatial attention and oculomotor control, such effects may plausibly involve the hemisphere opposite to the pain side. Attentional bias may be even more pronounced for learned pain-related signals, with increased attentional capture and delayed disengagement (Van Damme et al., 2006). Although TN is classified as neuropathic pain, individuals are prone to developing learned pain-related cues after repeated pairing with triggering factors, such as touch, chewing or cold wind. Such learned associations may further reinforce attentional bias and hypervigilance toward pain-related stimuli.

We also observed widespread reductions in non-focal, cross-network white matter connectivity, suggesting more extensive structural alterations beyond pain-related subnetworks and potentially contributing to broader network-level alterations. Previous diffusion MRI studies in TN have reported widespread FA reductions with a relatively broad and symmetric distribution, involving regions such as the corona radiata, internal capsule, optic radiation, and cerebellar peduncles (Zhang et al., 2018; DeSouza et al., 2014). these alterations have been reported to correlate with disease duration and pain severity, suggesting a link between network disruption and clinical severity (Liu et al., 2018). Prior studies using the Mini-Mental State Examination (MMSE) in patients with chronic pain revealed that these patients exhibited significantly lower MMSE scores than those with mild cognitive impairment (MCI), and that their cognitive deficits worsened with increasing pain severity (Moriya et al., 2024). One possible explanation is that progressive disruption of brain network connectivity may contribute to such cognitive changes. Existing literature has also proposed that chronic pain may be associated with accelerated brain aging and progressive cognitive decline (Zhao et al., 2025). Moreover, the reduction in white matter connectivity reflects a pathway in that persistent nociceptive input may trigger a degenerative process in the connectivity of the central nervous system, thus disrupting the integration of information between cortical–cortical and cortical–subcortical regions. Overall, these structural alterations in TN may reflect a combination of pathological change.

Previous imaging studies in TN have predominantly focused on the “core pain network”, identifying key regions such as the thalamus, insula, ACG, limbic system, and somatosensory and motor cortices (Xu et al., 2025; Wang Y. et al., 2015; Blatow et al., 2009). However, in the present study, graph-theoretical analysis revealed that both the nodal clustering coefficient and local efficiency of the cuneus on the affected side were significantly reduced, suggesting that its local fault tolerance and information integration capabilities were impaired. Surface-based and voxel-based morphometry studies have also reported reduced gray matter volume and cortical thickness in the cuneus in TN (Parise et al., 2014). Our findings further suggest that the cuneus on the pain side is structurally vulnerable, as evidenced by significant reductions in nodal clustering coefficient and local efficiency. Taken together, these findings indicate that the cuneus on the pain side exhibits localized network alterations at the nodal level, which may reflect region-specific alterations related to chronic pain.

Notably, exploratory stratification by BNI pain intensity score did not reveal significant differences between TN patients with BNI grades 3–4 and those with BNI grade 5 across the nodal abnormalities identified in the primary analysis, suggesting that these findings were not driven solely by the most severe pain subgroup. However, given the limited number of patients with BNI grade 5, this exploratory result should be interpreted cautiously, and larger studies are needed to further clarify whether nodal abnormalities vary according to pain severity.

From a clinical perspective, our findings support the notion that TN should be considered as a central network disorder, rather than being solely attributed to the compression of peripheral nerve roots. For patients, early intervention may reduce the frequency and intensity of painful episodes, potentially preventing the deterioration of white matter network connectivity. Treatment strategies should adopt a multi-dimensional approach, considering not only the alleviation of nociceptive input but also the assessment of cognitive and emotional states of mind. Consequently, we propose that DTI should be employed for the early evaluation of TN patients, monitoring white matter connectivity metrics such as network connectivity density. Future focus should be on closely monitoring the potential formation of pain subnetworks and promptly initiating a combination of interventions. Approaches, including pharmacotherapy, surgery, neuromodulation, and cognitive interventions, should be implemented with the goal of modifying disease trajectory.

This study has several limitations that should be considered. First, the human brain exhibits inherent left–right asymmetry in structural connectivity. Although a side-flipping strategy was applied to align patients with left and right-sided pain at the group level, residual effects related to hemispheric asymmetry in healthy individuals may still influence the results. In addition, this flipping procedure was applied only to TN patients and not to the HC group, which may introduce potential bias due to asymmetry in atlas-based parcellation. Second, the relatively modest sample size may limit the statistical power and generalizability of the findings. Although significant differences were observed, the possibility of reduced sensitivity to detect more subtle effects cannot be excluded. Therefore, the results should be interpreted with caution, and future studies with larger, independent cohorts are warranted to further validate these findings. Third, the relationship between graph-theoretical metrics and clinical variables (e.g., disease duration or pain severity) was not examined. Future studies with larger sample sizes are needed to further clarify the clinical relevance of these network alterations. Finally, diffusion data were acquired using a conventional 64-direction DTI protocol, which has limited ability to resolve complex fiber configurations such as crossing fibers compared with multi-shell diffusion imaging. Future studies using multi-shell diffusion imaging may improve the accuracy of fiber reconstruction and provide more reliable characterization of structural connectivity.

## 5 Conclusion

In this study, we utilized NBS and graph theoretical analysis to investigate alterations in the structural brain networks of patients with TN. We identified specific sub-networks associated with TN and revealed notable differences between the pain-affected and non-pain sides. These differences predominantly involved multiple brain networks, including those implicated in salience processing, sensory-motor integration, attention, and visual processing, alongside a generalized reduction in global network connectivity. Based on these observations, we propose that the identified sub-networks and nodes not only hold promise as potential biomarkers but should also be regarded as key elements in the clinical assessment and active management of TN patients.

## Data Availability

The original contributions presented in the study are included in the article/Supplementary material, further inquiries can be directed to the corresponding authors.

## Footnotes

1
http://www.nitrc.org/projects/panda

## Supplementary material

SUPPLEMENTARY FIGURE 1 Brain regions showing between-group differences in nodal graph-theoretical measures that did not survive FDR correction, visualized using the AAL-90 atlas. The brain regions (nodes) displayed in BrainNet Viewer represent exploratory between-group differences in network properties between trigeminal neuralgia (TN) patients and healthy controls (HCs). Intermediate-colored nodes indicate exploratory findings with large effect sizes (uncorrected *p* < 0.01 and |Cohen’s *d*| > 0.8), whereas light-colored nodes indicate regions with moderate effect sizes showing a statistical trend (uncorrected *p* < 0.05 and |Cohen’s d| > 0.5). Blue nodes represent HCs > TN, and red nodes represent TN > HCs.A) Nodal Shortest Path Length; B) Nodal Clustering Coefficient; C) Nodal Local Efficiency; D) Betweenness Centrality; E) Degree Centrality; F) Nodal Efficiency.

SUPPLEMENTARY FIGURE 2 Exploratory subgroup analysis of nodal measures stratified by BNI pain intensity score. TN patients were stratified into BNI grades 3–4 and BNI grade 5 and compared with HCs for nodal measures identified as significant in the primary nodal analysis. (A) IPL.pain, nodal shortest path length; (B) SFGdor.non_pain, nodal clustering coefficient; (C) CUN.pain, nodal clustering coefficient; (D) CUN.pain, nodal local efficiency. No significant difference was observed between the two TN subgroups across these nodal measures. P values shown are uncorrected exploratory results.
