# Supplementary figures and images for: Pain-side-specific alteration of structural networks in trigeminal neuralgia: a connectome analysis
Source: Front Neurosci. 2026 May 29;20:1794457. doi: 10.3389/fnins.2026.1794457 (PMC13270086; doi:10.3389/fnins.2026.1794457)

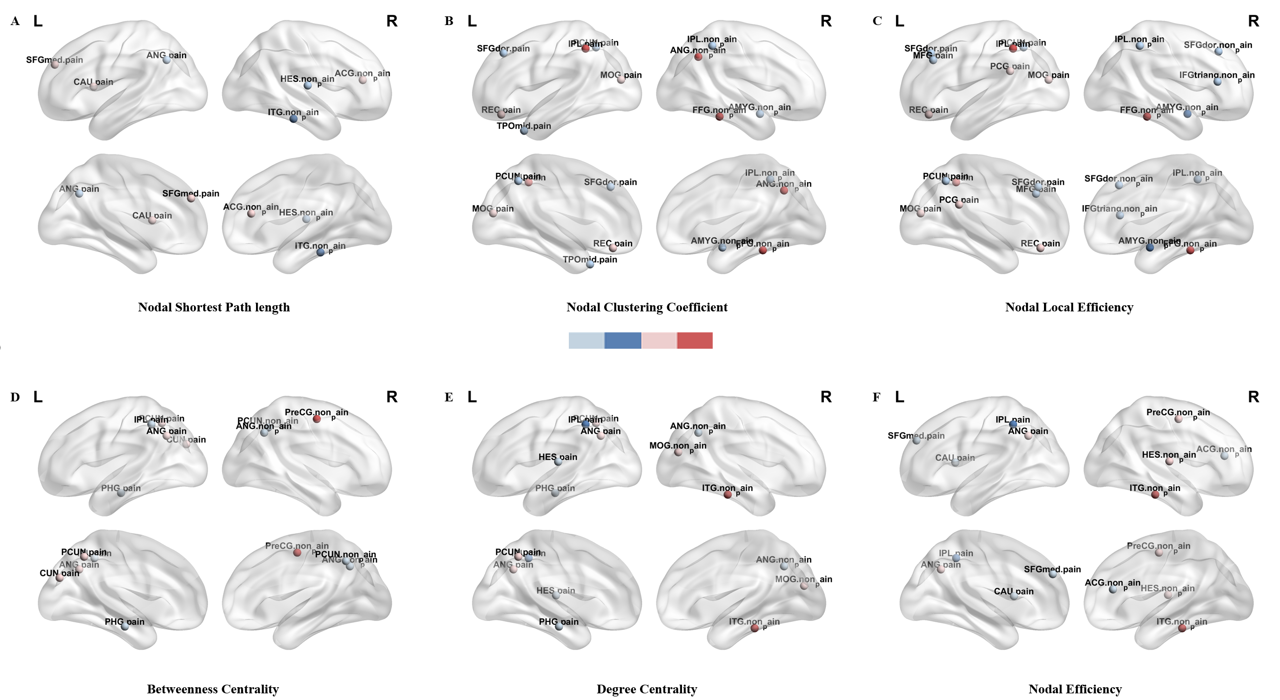

Supplement: SUPPLEMENTARY FIGURE 1 — Brain regions showing between-group differences in nodal graph-theoretical measures that did not survive FDR correction, visualized using the AAL-90 atlas. The brain regions (nodes) displayed in BrainNet Viewer represent exploratory between-group differences in network properties between trigeminal neuralgia (TN) patients and healthy controls (HCs). Intermediate-colored nodes indicate exploratory findings with large effect sizes (uncorrected p < 0.01 and |Cohen’s d| > 0.8), whereas light-colored nodes indicate regions with moderate effect sizes showing a statistical trend (uncorrected p < 0.05 and |Cohen’s d| > 0.5). Blue nodes represent HCs > TN, and red nodes represent TN > HCs.A) Nodal Shortest Path Length; B) Nodal Clustering Coefficient; C) Nodal Local Efficiency; D) Betweenness Centrality; E) Degree Centrality; F) Nodal Efficiency. [file Image_1.tif]

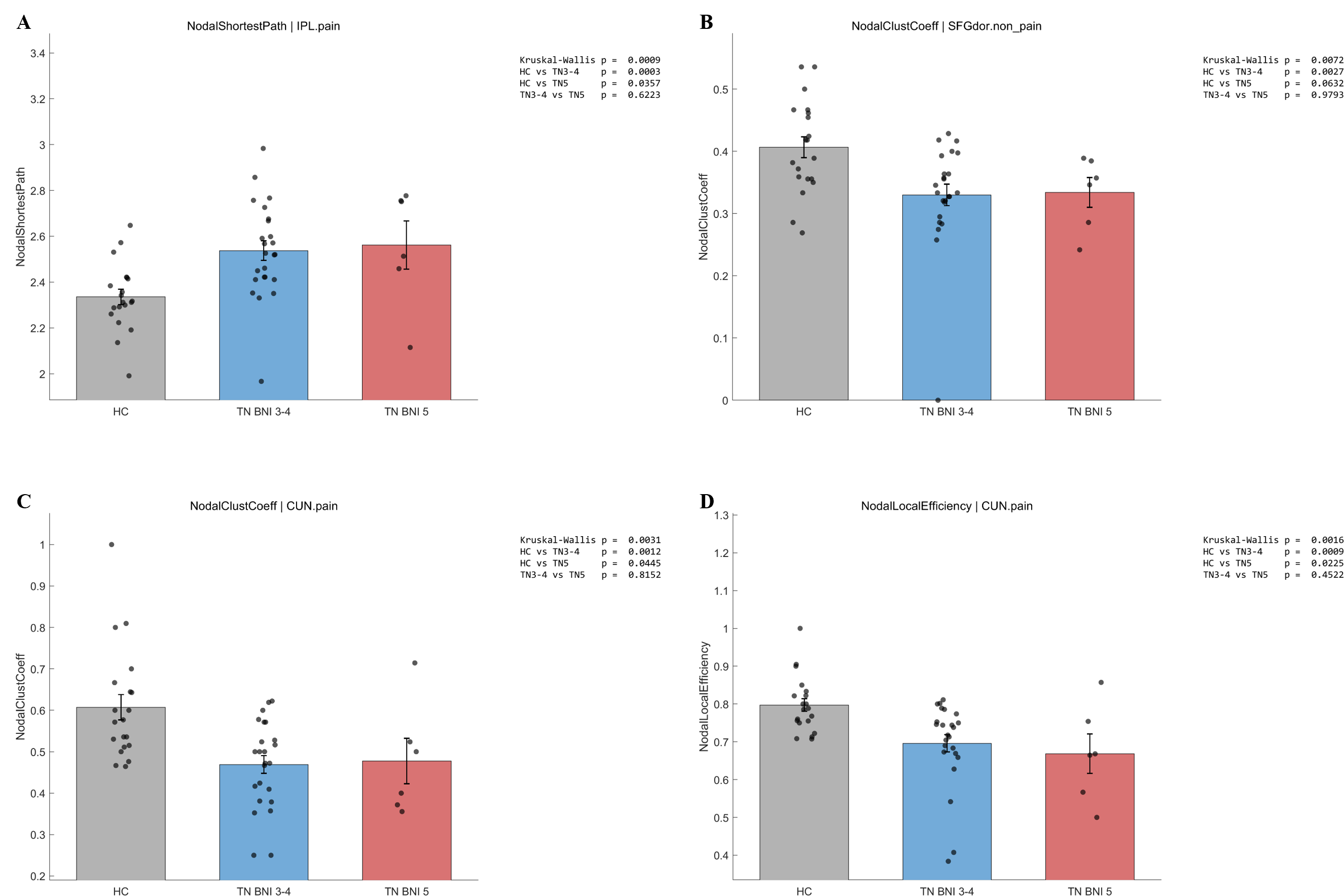

Supplement: SUPPLEMENTARY FIGURE 2 — Exploratory subgroup analysis of nodal measures stratified by BNI pain intensity score. TN patients were stratified into BNI grades 3–4 and BNI grade 5 and compared with HCs for nodal measures identified as significant in the primary nodal analysis. (A) IPL.pain, nodal shortest path length; (B) SFGdor.non_pain, nodal clustering coefficient; (C) CUN.pain, nodal clustering coefficient; (D) CUN.pain, nodal local efficiency. No significant difference was observed between the two TN subgroups across these nodal measures. P values shown are uncorrected exploratory results. [file Image_2.tif]

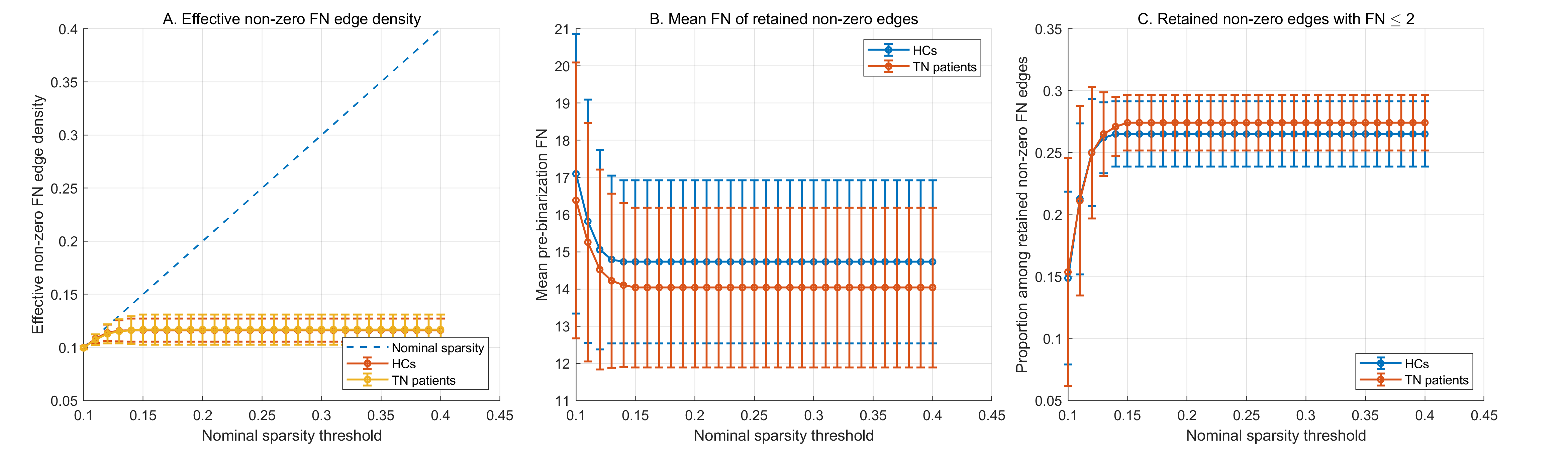

Supplement: Supplementary file 3 [file Image_3.tif]
