# Supplementary material for: Pain-side-specific alteration of structural networks in trigeminal neuralgia: a connectome analysis
Source: Front Neurosci. 2026 May 29;20:1794457. doi: 10.3389/fnins.2026.1794457 (PMC13270086; doi:10.3389/fnins.2026.1794457)
Supplement: Supplementary file 4 [file Table_1.docx]

**Supplementary Table 1. Barrow Neurological Institute (BNI) pain intensity score**

| **Score** | **Pain Description** |
| --- | --- |
| I | No trigeminal pain, no medication |
| II | Occasional pain, not requiring medication |
| III | Some pain, adequately controlled with medication |
| IV | Some pain, not adequately controlled with medication |
| V | Severe pain/no pain relief |

**Supplementary Table 2. The abbreviations of the 90 brain regions in AAL-90 atlas**

| **Abbreviations** | **Region** |
| --- | --- |
| PreCG | Precentral gyrus |
| SFGdor | Superior frontal gyrus, dorsolateral |
| ORBsup | Superior frontal gyrus, orbital part |
| MFG | Middle frontal gyrus |
| ORBmid | Middle frontal gyrus, orbital part |
| IFGoperc | Inferior frontal gyrus, opercular part |
| IFGtriang | Inferior frontal gyrus, triangular part |
| ORBinf | Inferior frontal gyrus, orbital part |
| ROL | Rolandic operculum |
| SMA | Supplementary motor area |
| OLF | Olfactory cortex |
| SFGmed | Superior frontal gyrus, medial |
| ORBMed | Superior frontal gyrus, medial orbital |
| REC | Gyrus rectus |
| INS | Insula |
| ACG | Anterior cingulate and paracingulate gyri |
| DCG | Median cingulate and paracingulate gyri |
| PCG | Posterior cingulate gyrus |
| HIP | Hippocampus |
| PHG | Para hippocampal gyrus |
| AMYG | Amygdala |
| CAL | Calcarine fissure and surrounding cortex |
| CUN | Cuneus |
| LING | Lingual gyrus |
| SOG | Superior occipital gyrus |
| MOG | Middle occipital gyrus |
| IOG | Inferior occipital gyrus |
| FFG | Fusiform gyrus |
| PoCG | Postcentral gyrus |
| SPG | Superior parietal gyrus |
| IPL | Inferior parietal lobule |
| SMG | Supramarginal gyrus |
| ANG | Angular gyrus |
| PCUN | Precuneus |
| PCL | Paracentral lobule |
| CAU | Caudate |
| PUT | Putamen |
| PAL | Pallidum |
| THA | Thalamus |
| HES | Heschl gyrus |
| STG | Superior temporal gyrus |
| TPOsup | Temporal pole (Superior) |
| MTG | Middle temporal gyrus |
| TPOmid | Temporal pole (Middle) |
| ITG | Inferior temporal |

**Supplementary Table 3. Results of global topological properties.**

| **Global topological properties** | **HCs (*n* = 20)**  **(Mean ± SD)** | **TN (*n* = 30)**  **(Mean ± SD)** | ***p* Value** |
| --- | --- | --- | --- |
| Global efficiency | 0.139 ± 0.005 | 0.138 ± 0.007 | 0.957 |
| Local efficiency | 0.206 ± 0.006 | 0.205 ± 0.007 | 0.754 |
| Clustering coefficient | 0.137 ± 0.006 | 0.137 ± 0.007 | 0.983 |
| Normalized clustering coefficient | 1.029 ± 0.111 | 1.026 ± 0.155 | 0.949 |
| Normalized characteristic path length | 0.332 ± 0.004 | 0.332 ± 0.007 | 0.822 |
| Characteristic path length | 0.651 ± 0.025 | 0.652 ± 0.033 | 0.891 |
| Small-worldness | 0.928 ± 0.091 | 0.926 ± 0.122 | 0.939 |

**Supplementary Table 4. Group comparisons of AUC-based nodal topological properties.**

| **Nodal Topological**  **Properties** | **HCs (*n* = 20)**  **(Mean ± SD)** | **TN (*n* = 30)**  **(Mean ± SD)** | ***p (unc.)*** | ***p_corr (FDR, q)*** | ***Cohen's d*** |
| --- | --- | --- | --- | --- | --- |
| **Nodal Shortest Path Length** |  |  |  |  |  |
| SFGmed.pain | 2.121 ± 0.180 | 2.242 ± 0.234 | 0.050 | 0.570 | 0.581 |
| ACG.non_pain | 2.128 ± 0.181 | 2.226 ± 0.163 | 0.047 | 0.571 | 0.571 |
| IPL.pain | 2.336 ± 0.149 | 2.542 ± 0.214 | <0.001 | 0.036 | 1.123 |
| ANG.pain | 2.552 ± 0.219 | 2.421 ± 0.180 | 0.023 | 0.436 | -0.656 |
| CAU.pain | 2.056 ± 0.104 | 2.144 ± 0.124 | 0.013 | 0.396 | 0.764 |
| HES.non_pain | 3.088 ± 0.255 | 2.917 ± 0.249 | 0.024 | 0.436 | -0.676 |
| ITG.non_pain | 2.493 ± 0.179 | 2.341 ± 0.193 | 0.007 | 0.315 | -0.813 |
| **Nodal Clustering Coefficient** |  |  |  |  |  |
| SFGdor.non_pain | 0.407 ± 0.075 | 0.331 ± 0.079 | 0.001 | 0.036 | -0.984 |
| REC.pain | 0.374 ± 0.138 | 0.452 ± 0.107 | 0.028 | 0.254 | 0.635 |
| AMYG.non_pain | 0.728 ± 0.187 | 0.611 ± 0.177 | 0.028 | 0.254 | -0.644 |
| CUN.pain | 0.607 ± 0.136 | 0.471 ± 0.109 | <0.001 | 0.018 | -1.106 |
| MOG.pain | 0.351 ± 0.069 | 0.425 ± 0.128 | 0.020 | 0.229 | 0.725 |
| FFG.non_pain | 0.450 ± 0.072 | 0.521 ± 0.089 | 0.005 | 0.110 | 0.876 |
| IPL.pain | 0.478 ± 0.088 | 0.616 ± 0.162 | 0.002 | 0.051 | 1.056 |
| IPL.non_pain | 0.679 ± 0.105 | 0.583 ± 0.139 | 0.012 | 0.161 | -0.781 |
| ANG.non_pain | 0.486 ± 0.070 | 0.568 ± 0.122 | 0.009 | 0.139 | 0.824 |
| PCUN.pain | 0.290 ± 0.070 | 0.250 ± 0.062 | 0.041 | 0.286 | -0.607 |
| TPOmid.pain | 0.549 ± 0.209 | 0.441 ± 0.146 | 0.039 | 0.286 | -0.597 |
| **Nodal Local Efficiency** |  |  |  |  |  |
| SFGdor.pain | 0.667 ± 0.093 | 0.589 ± 0.154 | 0.041 | 0.284 | -0.61 |
| SFGdor.non_pain | 0.663 ± 0.072 | 0.584 ± 0.133 | 0.01 | 0.156 | -0.738 |
| MFG.pain | 0.773 ± 0.107 | 0.695 ± 0.128 | 0.029 | 0.239 | -0.667 |
| IFGtriang.non_pain | 0.728 ± 0.077 | 0.648 ± 0.167 | 0.041 | 0.284 | -0.62 |
| REC.pain | 0.607 ± 0.199 | 0.697 ± 0.080 | 0.02 | 0.215 | 0.597 |
| PCG.pain | 0.636 ± 0.162 | 0.736 ± 0.141 | 0.028 | 0.239 | 0.656 |
| AMYG.non_pain | 0.858 ± 0.103 | 0.758 ± 0.138 | 0.007 | 0.13 | -0.821 |
| CUN.pain | 0.797 ± 0.074 | 0.691 ± 0.113 | <0.001 | 0.027 | -1.114 |
| MOG.pain | 0.618 ± 0.097 | 0.676 ± 0.100 | 0.045 | 0.289 | 0.588 |
| FFG.non_pain | 0.703 ± 0.055 | 0.749 ± 0.056 | 0.005 | 0.124 | 0.834 |
| IPL.pain | 0.723 ± 0.058 | 0.797 ± 0.096 | 0.004 | 0.108 | 0.941 |
| IPL.non_pain | 0.836 ± 0.057 | 0.780 ± 0.085 | 0.014 | 0.181 | -0.770 |
| PCUN.pain | 0.582 ± 0.061 | 0.531 ± 0.083 | 0.021 | 0.215 | -0.714 |
| **Degree Centrality** |  |  |  |  |  |
| PHG.pain | 9.500 ± 1.638 | 8.267 ± 1.818 | 0.019 | 0.351 | -0.713 |
| MOG.non_pain | 9.600 ± 2.854 | 11.933 ± 3.939 | 0.028 | 0.42 | 0.678 |
| IPL.pain | 8.600 ± 1.875 | 6.600 ± 2.358 | 0.002 | 0.18 | -0.939 |
| ANG.pain | 6.150 ± 1.565 | 7.500 ± 1.996 | 0.017 | 0.351 | 0.753 |
| ANG.non_pain | 9.150 ± 1.843 | 7.733 ± 2.490 | 0.038 | 0.445 | -0.647 |
| PCUN.pain | 15.550 ± 2.856 | 18.033 ± 3.746 | 0.017 | 0.351 | 0.746 |
| HES.pain | 2.900 ± 1.165 | 2.233 ± 0.898 | 0.040 | 0.445 | -0.641 |
| ITG.non_pain | 7.600 ± 2.010 | 9.567 ± 2.473 | 0.005 | 0.229 | 0.873 |
| **Betweenness Centrality** |  |  |  |  |  |
| PreCG.non_pain | 73.133 ± 39.835 | 127.219 ± 75.249 | 0.005 | 0.417 | 0.898 |
| PHG.pain | 38.613 ± 23.162 | 25.861 ± 15.616 | 0.026 | 0.444 | -0.646 |
| CUN.pain | 31.014 ± 34.369 | 81.639 ± 111.852 | 0.034 | 0.444 | 0.612 |
| IPL.pain | 34.942 ± 24.405 | 17.921 ± 28.994 | 0.031 | 0.444 | -0.635 |
| ANG.pain | 10.175 ± 10.761 | 19.149 ± 17.519 | 0.048 | 0.459 | 0.617 |
| ANG.non_pain | 34.022 ± 30.276 | 16.891 ± 18.777 | 0.011 | 0.417 | -0.68 |
| PCUN.pain | 251.459 ± 126.365 | 376.052 ± 191.050 | 0.014 | 0.417 | 0.769 |
| PCUN.non_pain | 437.797 ± 160.851 | 341.319 ± 149.181 | 0.034 | 0.444 | -0.622 |
| **Nodal Efficiency** |  |  |  |  |  |
| PreCG.non_pain | 0.455±0.020 | 0.473±0.035 | 0.037 | 0.498 | 0.66 |
| SFGmed.pain | 0.475±0.038 | 0.450±0.044 | 0.049 | 0.547 | -0.584 |
| ACG.non_pain | 0.474±0.038 | 0.452±0.033 | 0.039 | 0.498 | -0.604 |
| IPL.pain | 0.430±0.028 | 0.396±0.036 | 0.001 | 0.081 | -1.054 |
| ANG.pain | 0.394±0.032 | 0.415±0.029 | 0.022 | 0.468 | 0.679 |
| CAU.pain | 0.488±0.025 | 0.468±0.027 | 0.016 | 0.468 | -0.745 |
| HES.non_pain | 0.326±0.026 | 0.345±0.030 | 0.026 | 0.468 | 0.69 |
| ITG.non_pain | 0.403±0.030 | 0.430±0.034 | 0.008 | 0.369 | 0.831 |
