# Supplementary material for: Pain-side-specific alteration of structural networks in trigeminal neuralgia: a connectome analysis
Source: Front Neurosci. 2026 May 29;20:1794457. doi: 10.3389/fnins.2026.1794457 (PMC13270086; doi:10.3389/fnins.2026.1794457)
Supplement: Supplementary file 5 [file Data_Sheet_1.docx]

**Supplementary Material**

**Supplementary Methods 1: Raw fiber-number (FN) characteristics of retained edges across nominal sparsity thresholds**

To further characterize the graph-thresholding procedure, we examined the original PANDA fiber-number matrices before binarization. For each participant, the 90 × 90 AAL FN matrix represented raw streamline count values between predefined brain regions, which were treated as network nodes. After excluding diagonal elements and using the upper triangular matrix, 4,005 possible undirected node-to-node entries were obtained. At each nominal proportional sparsity threshold from 0.10 to 0.40, node-to-node entries were ranked in descending order according to their raw FN values, and the strongest entries were nominally retained before binarization. We summarized the pre-binarization FN characteristics of these retained entries, including the number of retained entries with FN > 0, effective non-zero FN edge density, cutoff FN, mean FN, total FN, and the proportions of low-FN retained non-zero edges.

The pre-binarization FN characteristics of retained node-to-node entries across nominal sparsity thresholds are provided in **Supplementary Table S5**. The proportions of low-FN retained non-zero edges are provided in **Supplementary Table S6**. The relationship between nominal sparsity thresholds and pre-binarization FN characteristics is visualized in **Supplementary Figure S3**.

**Supplementary Table S5. Pre-binarization fiber-number (FN) characteristics of retained node-to-node entries across nominal sparsity thresholds.**

| **Group** | **Nominal sparsity** | **Nominal retained edges** | **Retained non-zero**  **FN edges** | **Effective non-zero**  **FN edge density** | **Cutoff FN, all nominally retained entries** | **Mean FN among retained non-zero edges** | **Total FN among retained non-zero edges** |
| --- | --- | --- | --- | --- | --- | --- | --- |
| HCs | 0.10 | 401 | 399.850 ± 5.143 | 0.100 ± 0.001 | 1.450 ± 0.759 | 17.097 ± 3.758 | 6841.1 ± 1525.6 |
| HCs | 0.11 | 441 | 433.700 ± 16.432 | 0.108 ± 0.004 | 0.800 ± 0.616 | 15.820 ± 3.276 | 6883.2 ± 1546.6 |
| HCs | 0.12 | 481 | 455.500 ± 31.139 | 0.114 ± 0.008 | 0.350 ± 0.489 | 15.057 ± 2.678 | 6907.2 ± 1566.2 |
| HCs | 0.20 | 801 | 465.200 ± 43.475 | 0.116 ± 0.011 | 0.000 ± 0.000 | 14.730 ± 2.191 | 6916.9 ± 1580.2 |
| HCs | 0.30 | 1202 | 465.200 ± 43.475 | 0.116 ± 0.011 | 0.000 ± 0.000 | 14.730 ± 2.191 | 6916.9 ± 1580.2 |
| HCs | 0.40 | 1602 | 465.200 ± 43.475 | 0.116 ± 0.011 | 0.000 ± 0.000 | 14.730 ± 2.191 | 6916.9 ± 1580.2 |
| TN | 0.10 | 401 | 398.700 ± 6.358 | 0.100 ± 0.002 | 1.300 ± 0.877 | 16.385 ± 3.712 | 6539.2 ± 1513.2 |
| TN | 0.11 | 441 | 429.570 ± 20.801 | 0.107 ± 0.005 | 0.800 ± 0.664 | 15.258 ± 3.204 | 6580.6 ± 1537.9 |
| TN | 0.12 | 481 | 451.770 ± 35.628 | 0.113 ± 0.009 | 0.400 ± 0.563 | 14.523 ± 2.688 | 6606.7 ± 1557.2 |
| TN | 0.20 | 801 | 467.800 ± 57.069 | 0.117 ± 0.014 | 0.000 ± 0.000 | 14.040 ± 2.149 | 6623.4 ± 1580.0 |
| TN | 0.30 | 1202 | 467.800 ± 57.069 | 0.117 ± 0.014 | 0.000 ± 0.000 | 14.040 ± 2.149 | 6623.4 ± 1580.0 |
| TN | 0.40 | 1602 | 467.800 ± 57.069 | 0.117 ± 0.014 | 0.000 ± 0.000 | 14.040 ± 2.149 | 6623.4 ± 1580.0 |

Values are presented as mean ± SD across participants. FN, fiber number.

Each AAL90 matrix contained 4,005 possible undirected node-to-node entries after excluding diagonal elements and using the upper triangular matrix. Nominally retained node-to-node entries indicate the number of entries retained according to the nominal proportional sparsity threshold. Retained non-zero FN edges indicate retained entries with FN > 0. Effective non-zero FN edge density was calculated as the number of retained non-zero FN edges divided by 4,005. Cutoff FN was defined as the FN value of the last nominally retained entry after ranking all node-to-node entries by raw FN. Therefore, cutoff FN could reach 0 when the nominal sparsity exceeded the native non-zero FN edge density. Mean FN and total FN were calculated only among retained non-zero FN edges.

**Supplementary Table S6. Proportions of low-FN retained non-zero edges across nominal sparsity thresholds.**

| Group | Nominal sparsity | FN = 1 among retained non-zero FN edges | FN ≤ 2 among retained non-zero FN edges | FN ≤ 3 among retained non-zero FN edges |
| --- | --- | --- | --- | --- |
| HCs | 0.10 | 0.050 ± 0.054 | 0.149 ± 0.070 | 0.225 ± 0.066 |
|  | 0.11 | 0.108 ± 0.056 | 0.213 ± 0.061 | 0.286 ± 0.052 |
|  | 0.12 | 0.146 ± 0.041 | 0.250 ± 0.043 | 0.320 ± 0.034 |
|  | 0.20 | 0.163 ± 0.019 | 0.265 ± 0.026 | 0.333 ± 0.023 |
|  | 0.30 | 0.163 ± 0.019 | 0.265 ± 0.026 | 0.333 ± 0.023 |
|  | 0.40 | 0.163 ± 0.019 | 0.265 ± 0.026 | 0.333 ± 0.023 |
| TN | 0.10 | 0.061 ± 0.067 | 0.154 ± 0.092 | 0.239 ± 0.092 |
|  | 0.11 | 0.109 ± 0.063 | 0.211 ± 0.077 | 0.295 ± 0.070 |
|  | 0.12 | 0.144 ± 0.055 | 0.250 ± 0.053 | 0.330 ± 0.049 |
|  | 0.20 | 0.171 ± 0.018 | 0.274 ± 0.022 | 0.351 ± 0.023 |
|  | 0.30 | 0.171 ± 0.018 | 0.274 ± 0.022 | 0.351 ± 0.023 |
|  | 0.40 | 0.171 ± 0.018 | 0.274 ± 0.022 | 0.351 ± 0.023 |

Values are presented as mean ± SD across participants. FN, fiber number.

Proportions were calculated among retained non-zero FN edges. FN = 1, FN ≤ 2, and FN ≤ 3 were used to describe the proportion of low streamline count edges retained before binarization.

**Supplementary Figure S3. Relationship between nominal sparsity thresholds and pre-binarization fiber-number (FN) characteristics.**

(A) Effective non-zero FN edge density across nominal sparsity thresholds. The dashed line represents the nominal sparsity threshold, and the solid lines represent the effective non-zero FN edge density in HCs and TN patients.

(B) Mean pre-binarization FN values of retained non-zero edges across nominal sparsity thresholds.

(C) Proportion of retained non-zero edges with FN ≤ 2 across nominal sparsity thresholds.

Data are presented as mean ± SD. FN values were extracted from the original PANDA fiber-number matrices before binarization. HCs, healthy controls; TN, trigeminal neuralgia.

**Supplementary Methods 2: Raw fiber-number (FN) characteristics of NBS suprathreshold edges**

In the NBS analysis, edge-wise between-group *t* statistics were calculated for all node-to-node connections. A primary threshold of *t* = 2.1 was applied to this edge-wise statistical matrix to identify suprathreshold edges. These suprathreshold edges were then grouped into connected components, and component-level statistical inference was performed using permutation testing, as described in the main Methods section.

Because the NBS primary threshold was applied to edge-wise *t* statistics rather than to raw FN values, it did not represent a fixed streamline count cutoff. To characterize the streamline count profile of the suprathreshold edges, the edge-wise *t*-statistic matrix generated by NBS was matched to the original PANDA FN matrices. For each suprathreshold node-to-node edge, the corresponding raw FN values were extracted from each participant’s original FN matrix.

This procedure was performed separately for the TN > HCs and HCs > TN contrasts. For each contrast, suprathreshold edges identified at the primary threshold of *t* = 2.1 were listed according to their connected component membership before component-level permutation inference. Raw FN values were summarized separately for TN patients and HCs as mean ± SD. The raw FN characteristics of suprathreshold edges in the TN > HCs contrast are provided in **Supplementary Table S7**, and the corresponding results for the HCs > TN contrast are provided in **Supplementary Table S8**. The final NBS subnetworks surviving component-level permutation testing are reported in the main manuscript, whereas the supplementary edge-level summaries provide the raw FN characteristics of suprathreshold edges identified before component-level permutation inference.

**Supplementary Table S7. Raw fiber-number (FN) values for suprathreshold node-to-node edges identified in the TN > HCs contrast at the NBS primary threshold of t = 2.1 before component-level permutation inference.**

| **Component** | **Node1** | **Node2** | **HCs** | **TN** | **Edge-wise**  **t statistic** |
| --- | --- | --- | --- | --- | --- |
| 1 | SFGdor.pain | ACG.pain | 0.000 ± 0.000 | 2.300 ± 3.631 | 2.82 |
|  | ACG.pain | DCG.pain | 32.450 ± 18.392 | 45.300 ± 19.839 | 2.31 |
|  | ACG.pain | PCUN.pain | 3.600 ± 4.512 | 10.533 ± 13.310 | 2.24 |
|  | PoCG.pain | PCUN.pain | 0.250 ± 0.550 | 4.400 ± 7.005 | 2.63 |
|  | PoCG.pain | PCL.pain | 1.150 ± 1.531 | 7.667 ± 8.519 | 3.37 |
|  | PCUN.pain | PCL.pain | 5.350 ± 5.224 | 12.300 ± 10.920 | 2.65 |
| 2 | ORBsup.pain | TPOsup.pain | 7.700 ± 9.217 | 18.700 ± 20.395 | 2.26 |
|  | STG.pain | TPOsup.pain | 16.750 ± 13.062 | 31.900 ± 22.913 | 2.68 |
|  | ORBsup.pain | TPOmid.pain | 0.650 ± 2.033 | 4.267 ± 5.813 | 2.67 |
| 3 | MOG.nonpain | FFG.nonpain | 4.600 ± 5.471 | 15.700 ± 18.874 | 2.55 |
|  | MOG.nonpain | SPG.nonpain | 8.150 ± 12.584 | 24.567 ± 22.726 | 2.94 |
|  | MOG.nonpain | IPL.nonpain | 0.000 ± 0.000 | 9.600 ± 13.589 | 3.15 |
| 4 | PreCG.pain | ANG.pain | 0.800 ± 1.576 | 13.733 ± 23.647 | 2.43 |
|  | SOG.pain | ANG.pain | 0.000 ± 0.000 | 4.467 ± 7.431 | 2.68 |
| 5 | HIP.nonpain | TPOsup.nonpain | 1.550 ± 2.164 | 5.300 ± 7.236 | 2.24 |
|  | AMYG.nonpain | TPOsup.nonpain | 9.300 ± 6.906 | 17.700 ± 12.991 | 2.65 |
| 6 | PreCG.nonpain | ITG.nonpain | 0.050 ± 0.224 | 4.800 ± 9.834 | 2.15 |
| 7 | SFGdor.nonpain | ORBmid.nonpain | 0.050 ± 0.224 | 0.867 ± 1.167 | 3.08 |
| 8 | SMA.pain | PCL.nonpain | 1.950 ± 8.721 | 11.567 ± 17.712 | 2.25 |
| 9 | OLF.pain | REC.pain | 8.500 ± 5.708 | 12.833 ± 5.363 | 2.73 |
| 10 | REC.nonpain | ACG.nonpain | 0.050 ± 0.224 | 1.033 ± 2.059 | 2.12 |
| 11 | HIP.pain | THA.pain | 15.500 ± 12.142 | 25.500 ± 16.182 | 2.35 |

**Note:** FN values were extracted from the original PANDA FN matrices for each suprathreshold node-to-node edge and are presented as mean ± SD across participants within each group. Suprathreshold components were identified using the NBS primary threshold of *t* = 2.1 before component-level permutation inference. This table is provided to clarify how the NBS primary threshold mapped onto the underlying streamline count data. Component-level permutation-significant subnetworks are reported in the main manuscript.

**Supplementary Table S8. Raw fiber-number (FN) values for suprathreshold node-to-node edges identified in the HCs > TN contrast at the NBS primary threshold of t = 2.1 before component-level permutation inference.**

| **Component** | **Node1** | **Node2** | **HCs** | **TN** | **Edge-wise**  **t statistic** |
| --- | --- | --- | --- | --- | --- |
| 1 | MFG.nonpain | ORBinf.nonpain | 2.000 ± 4.600 | 0.033 ± 0.183 | 2.351 |
|  | ORBinf.pain | REC.pain | 12.250 ± 12.135 | 6.033 ± 6.122 | 2.394 |
|  | OLF.nonpain | ACG.pain | 1.900 ± 3.478 | 0.400 ± 0.814 | 2.281 |
|  | REC.pain | ACG.pain | 4.550 ± 5.708 | 0.733 ± 1.388 | 3.526 |
|  | MFG.nonpain | ACG.nonpain | 0.850 ± 2.134 | 0.000 ± 0.000 | 2.193 |
|  | SFGmed.pain | ACG.nonpain | 11.750 ± 19.147 | 2.200 ± 5.635 | 2.581 |
|  | ACG.nonpain | DCG.pain | 1.300 ± 3.147 | 0.033 ± 0.183 | 2.210 |
|  | PHG.pain | AMYG.pain | 16.400 ± 9.544 | 9.267 ± 9.555 | 2.587 |
|  | CAL.pain | LING.nonpain | 1.900 ± 3.354 | 0.267 ± 0.740 | 2.587 |
|  | AMYG.pain | SOG.pain | 0.150 ± 0.366 | 0.000 ± 0.000 | 2.254 |
|  | CAL.pain | SOG.pain | 19.250 ± 11.867 | 11.767 ± 9.104 | 2.520 |
|  | IFGoperc.pain | SPG.pain | 0.250 ± 0.639 | 0.000 ± 0.000 | 2.155 |
|  | AMYG.pain | SPG.pain | 0.250 ± 0.639 | 0.000 ± 0.000 | 2.155 |
|  | SPG.pain | SPG.nonpain | 1.050 ± 2.212 | 0.100 ± 0.403 | 2.307 |
|  | ROL.nonpain | ANG.nonpain | 2.350 ± 4.804 | 0.400 ± 1.102 | 2.150 |
|  | SOG.nonpain | ANG.nonpain | 9.500 ± 9.736 | 3.800 ± 6.703 | 2.455 |
|  | SOG.nonpain | PCUN.nonpain | 14.550 ± 8.703 | 8.233 ± 5.594 | 3.130 |
|  | PoCG.nonpain | PCUN.nonpain | 4.500 ± 4.383 | 1.633 ± 4.056 | 2.371 |
|  | SPG.pain | PCUN.nonpain | 14.750 ± 22.332 | 3.033 ± 10.572 | 2.494 |
|  | PoCG.nonpain | PCL.nonpain | 11.850 ± 8.756 | 4.933 ± 7.423 | 3.004 |
|  | OLF.nonpain | CAU.pain | 2.150 ± 2.661 | 0.800 ± 1.215 | 2.433 |
|  | ACG.pain | CAU.pain | 10.000 ± 7.049 | 5.633 ± 5.857 | 2.380 |
|  | SFGmed.pain | CAU.nonpain | 1.700 ± 3.045 | 0.067 ± 0.254 | 2.938 |
|  | ACG.nonpain | PUT.nonpain | 0.700 ± 1.750 | 0.000 ± 0.000 | 2.202 |
|  | SPG.nonpain | PUT.nonpain | 17.350 ± 22.528 | 6.400 ± 8.728 | 2.414 |
|  | CAU.pain | PUT.nonpain | 0.400 ± 0.995 | 0.000 ± 0.000 | 2.214 |
|  | CAU.nonpain | PUT.nonpain | 21.600 ± 16.210 | 11.700 ± 7.966 | 2.874 |
|  | PUT.nonpain | TPOsup.nonpain | 10.000 ± 15.980 | 1.567 ± 2.800 | 2.840 |
|  | STG.nonpain | TPOsup.nonpain | 35.000 ± 18.169 | 24.667 ± 15.323 | 2.168 |
|  | PUT.nonpain | TPOmid.nonpain | 2.000 ± 2.428 | 0.367 ± 0.999 | 3.302 |
| 2 | PreCG.pain | PoCG.pain | 102.500 ± 47.637 | 75.633 ± 36.172 | 2.265 |
|  | ROL.pain | PoCG.pain | 21.900 ± 11.447 | 11.467 ± 10.966 | 3.239 |
|  | PoCG.pain | IPL.pain | 45.650 ± 31.721 | 24.900 ± 24.654 | 2.598 |
| 3 | SFGdor.pain | IFGtriang.pain | 12.400 ± 20.922 | 1.000 ± 2.133 | 2.977 |
|  | ORBmid.pain | IFGtriang.pain | 2.550 ± 3.086 | 0.567 ± 1.813 | 2.864 |
| 4 | HIP.pain | HIP.nonpain | 0.400 ± 0.821 | 0.033 ± 0.183 | 2.372 |
|  | HIP.nonpain | THA.nonpain | 31.550 ± 22.156 | 18.200 ± 13.363 | 2.660 |
| 5 | LING.pain | MOG.pain | 6.100 ± 6.553 | 2.467 ± 3.511 | 2.546 |
|  | MOG.pain | FFG.pain | 23.300 ± 24.288 | 10.133 ± 14.802 | 2.385 |
|  | SMG.pain | ITG.pain | 2.850 ± 5.724 | 0.300 ± 1.643 | 2.312 |
| 6 | TPOsup.pain | ITG.pain | 0.750 ± 1.209 | 0.067 ± 0.254 | 3.014 |
| 7 | ORBsup.nonpain | ORBsupmed.nonpain | 4.650 ± 3.843 | 1.700 ± 2.136 | 3.484 |
| 8 | IFGoperc.nonpain | SMA.pain | 2.650 ± 6.063 | 0.167 ± 0.648 | 2.236 |
| 9 | SMA.nonpain | DCG.nonpain | 66.200 ± 40.594 | 43.733 ± 24.484 | 2.444 |
| 10 | SFGmed.nonpain | ORBsupmed.pain | 8.150 ± 13.295 | 0.833 ± 1.599 | 2.997 |
| 11 | REC.nonpain | AMYG.nonpain | 0.800 ± 1.735 | 0.033 ± 0.183 | 2.413 |
| 12 | CAL.nonpain | CUN.nonpain | 89.300 ± 35.157 | 67.667 ± 31.037 | 2.290 |
| 13 | IPL.nonpain | SMG.nonpain | 33.500 ± 23.650 | 20.633 ± 16.645 | 2.260 |

**Note:** FN values were extracted from the original PANDA FN matrices for each suprathreshold node-to-node edge and are presented as mean ± SD across participants within each group. Suprathreshold components were identified using the NBS primary threshold of *t* = 2.1 before component-level permutation inference. This table is provided to clarify how the NBS primary threshold mapped onto the underlying streamline count data. Component-level permutation-significant subnetworks are reported in the main manuscript.
